# Supplementary material for: Differential Effects of Extracellular Vesicles of Lineage-Specific Human Pluripotent Stem Cells on the Cellular Behaviors of Isogenic Cortical Spheroids
Source: Cells. 2019 Aug 28;8(9):993. doi: 10.3390/cells8090993 (PMC6770916; doi:10.3390/cells8090993)
Supplement: Supplementary file 1 [file cells-08-00993-s001.pdf]

Supplementary Materials

Differential Effects of Extracellular Vesicles of Lineage-specific Human Pluripotent Stem Cells on Cellular Behaviors of Isogenic Cortical Spheroids

Mark Marzano[1], Julie Bejoy[1], Mujeeb Cheerathodi[2], Li Sun[2], Sara York[2], Jing Zhao[3], Takahisa Kanekiyo[3], Guojun Bu[3], David Meckes[2], Yan Li[1, *]

[1]Department of Chemical and Biomedical Engineering; FAMU-FSU College of Engineering; Florida State University; Tallahassee, FL USA

[2]Department of Biomedical Sciences, College of Medicine, Florida State University, Tallahassee, Florida, USA

[3]Department of Neuroscience, Alzheimer's Disease Research Center, Mayo Clinic, Jacksonville, FL USA

Supplementary Figure S1. Flow cytometry analysis of undifferentiated iPSCs and neural progenitors. (A) Two-color flow cytometry analysis of LIVE/DEAD assay stained iPSK3 cells. The plot showed the percentage of viable cells of undifferentiated iPSK3 cells. (B) Flow cytometry analysis of β-tubulin III at day 24 of neural differentiation. The SSC-FSC plot shows the gated cell population. The histogram shows the positive cell population. Black line: negative control; Red line: marker of interest.

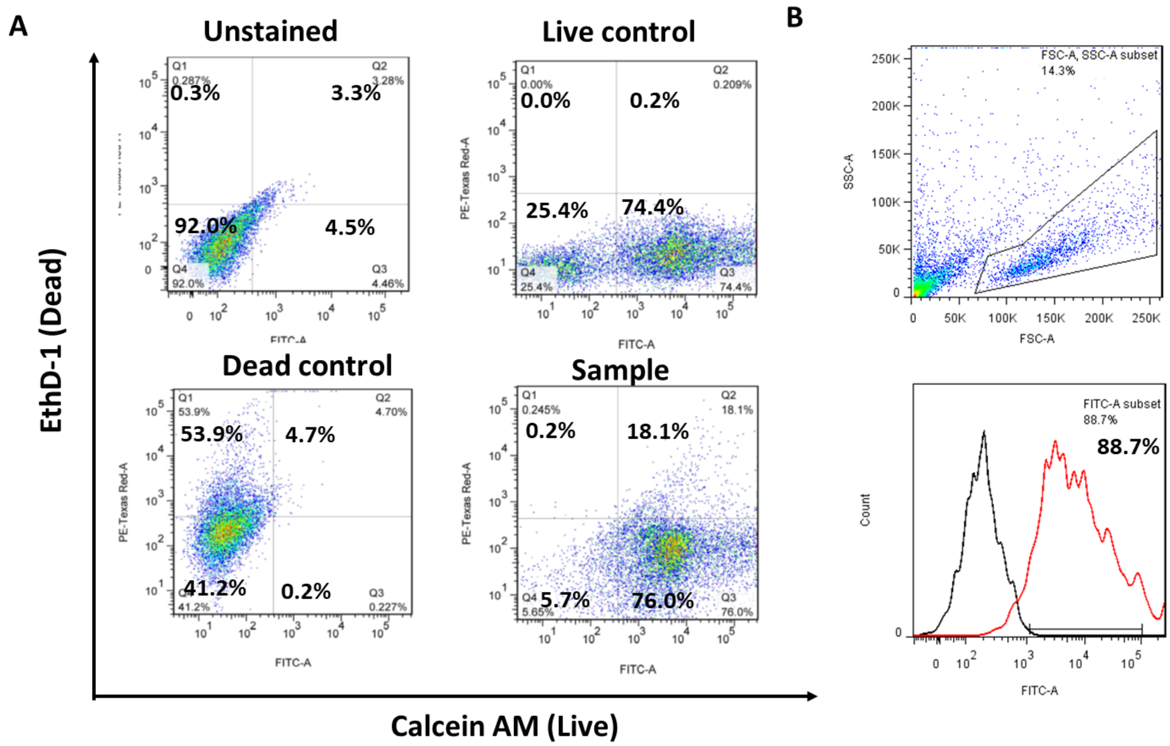

Supplementary Figure S2. Flow cytometry analysis of cardiac progenitors. Flow cytometry analysis of α-actinin was performed for day 20 of cardiac differentiation of iPSK3 cells. The dot plots were used to present the positive cell population.

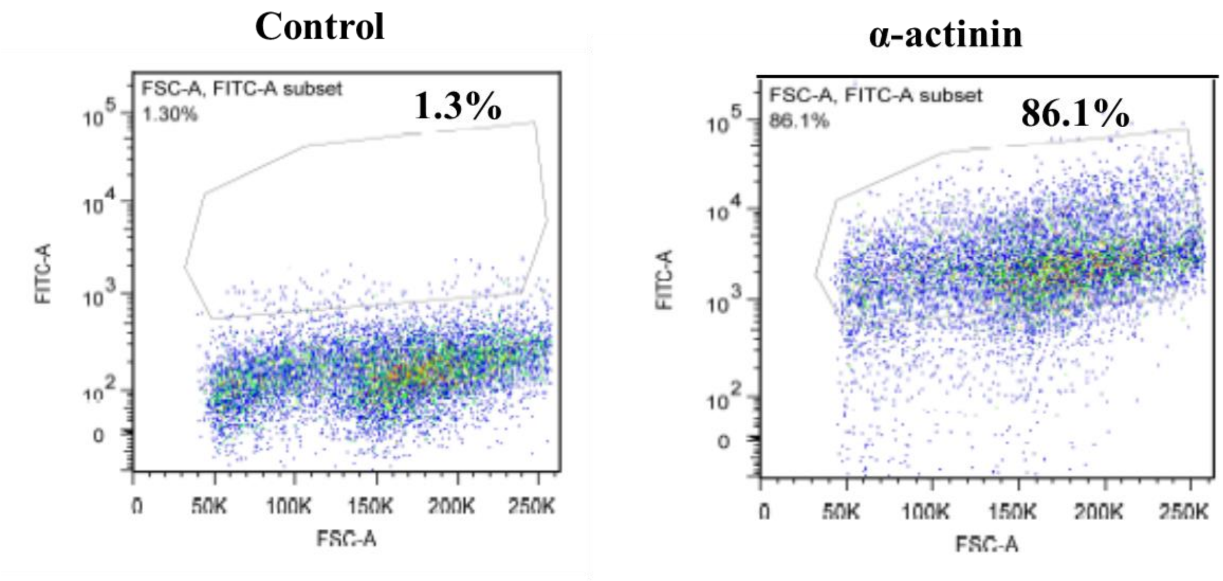

Supplementary Figure S3. Quantification of Western blot bands for Figure 3A(ii)a. (i) Alix; (ii) CD63; (iii) TSG101; (iv) Hsc70; (v) Western Blot results showing the absence of Calnexin expression in the derived EVs. Calnexin is a negative marker of EVs (but present in cell lysate). EV markers TSG101 and CD81 are present in both EVs and whole cell lysate (WCE) for iPSK3 and Ecto groups.

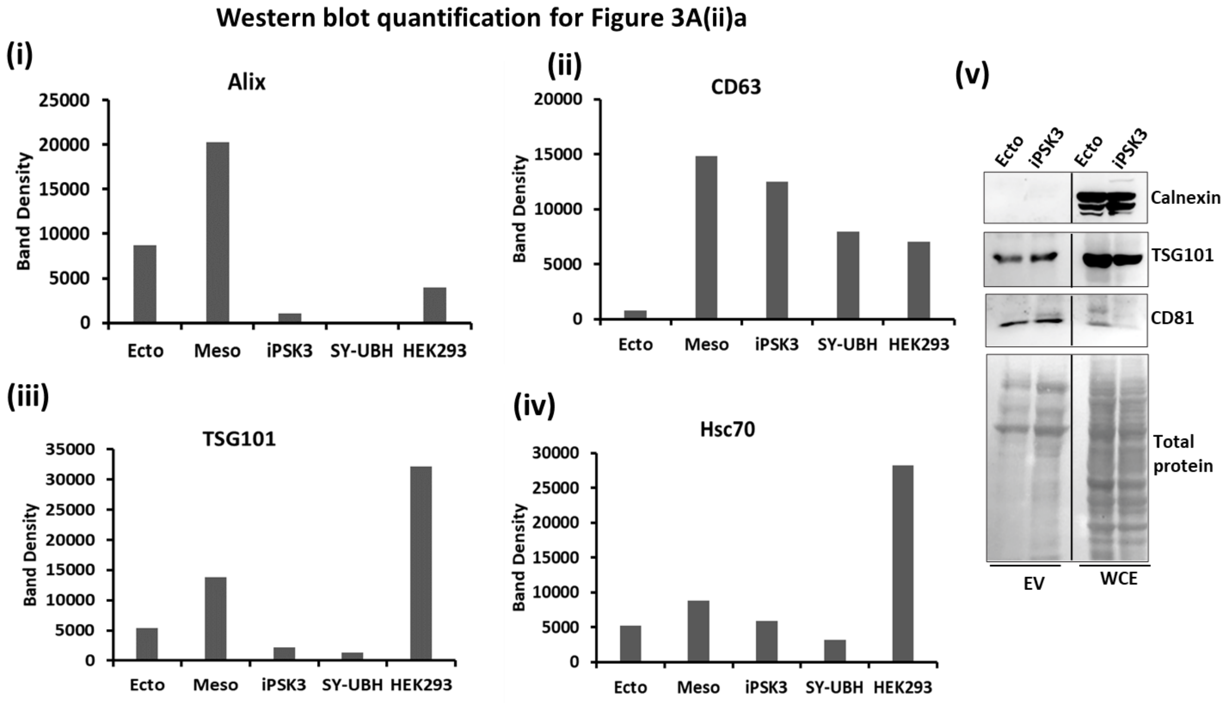

Supplementary Figure S4. Confirmation of the equal protein loading for Figure 3A(ii)a. The total protein stain used Ponceau S from Sigma (P7170-1L). Blots were incubated for 5 minutes with rocking prior to washing with water and imaging. EV: extracellular vesicles. WCE: whole cell extracts. NR: non-reducing format.

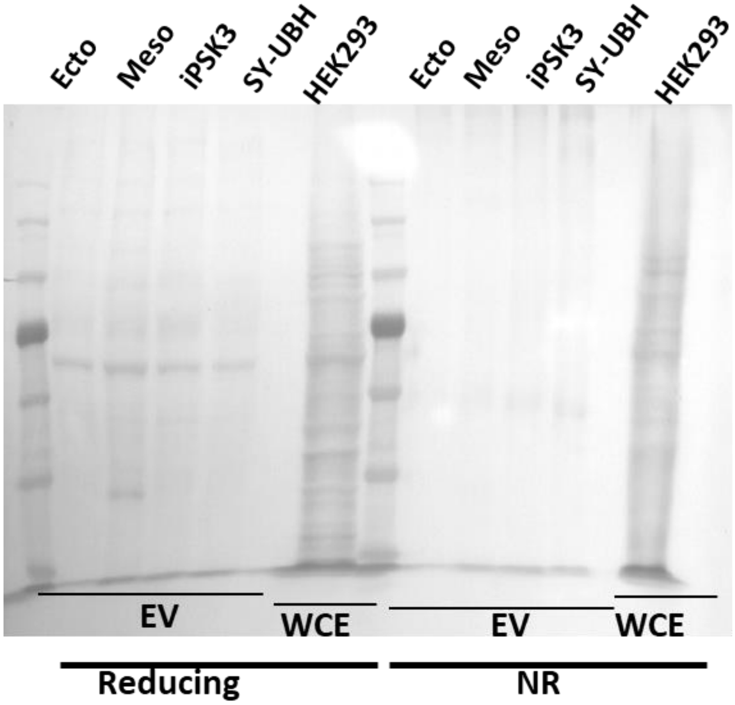

Supplementary Figure S5. Flow cytometry analysis of iPSC-derived EVs. Forward scatter and side scatter plots were shown to confirm the presence of iPSC-EVs compared to PBS control.

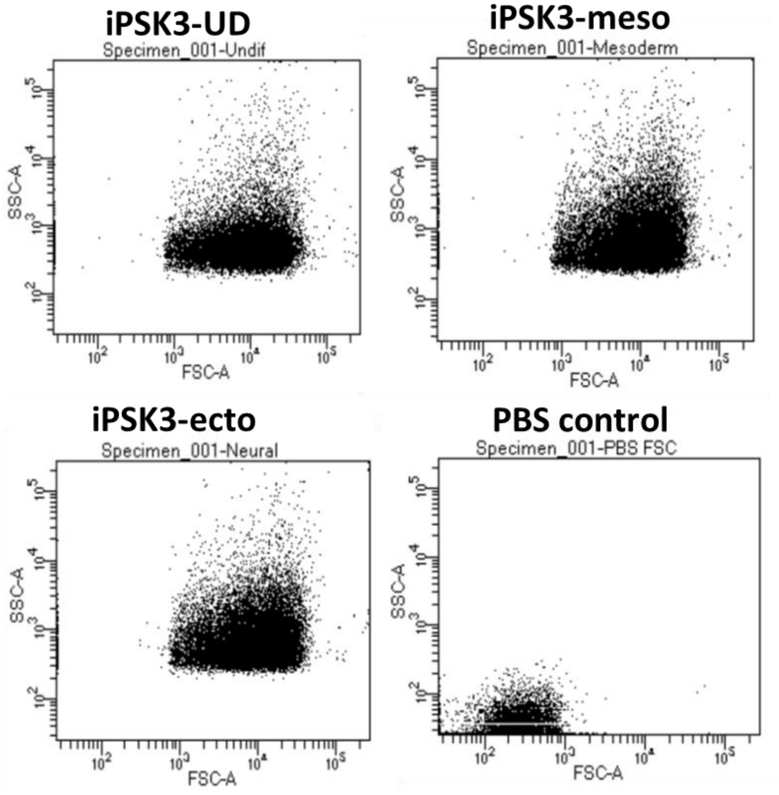

Supplementary Figure S6. Flow cytometry analysis of LIVE/DEAD assay (supporting data for Figure 6). (A) Assay was performed for cortical spheroid outgrowth treated with Aβ42 oligomers or SY-UBH derived-EV in addition to Aβ42 oligomers. (B) Viability determined by flow cytometry analysis in a separate experiment. (C) Viability determined by flow cytometry analysis in another separate experiment to obtain the result for iPSK3-UD group. In this experiment, the iPSK3-UD group had the increased viability, but not to the level of the Control group.

A

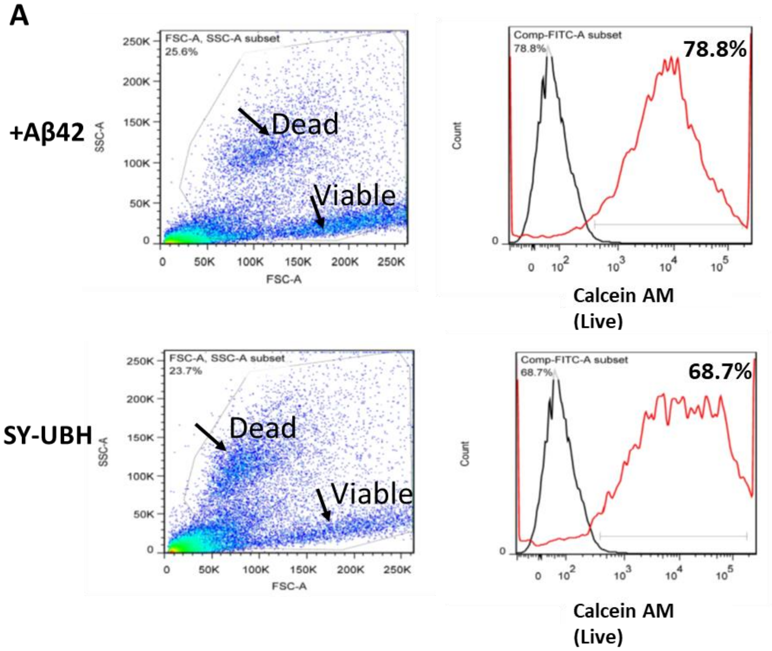

B

Groups	Viability by Flow Cytometry (%)	Comments
Control	89.7	
iPSK3-ecto	84.8	
iPSK3-meso	92.6	
iPSK3-UD	NA	low event
SY-UBH	57.0	low event
+Aβ42	77.2	

C

Groups	Viability by Flow Cytometry (%)
Control	80.9
iPSK3-UD	71.6
+Aβ42	66.6

Supplementary Figure S7. miRNA isolation and detection from iPSC-EVs. (A) Amplification Ct curve for the cDNA made from the miRNA isolated from three types of iPSC-EVs. (B) Melt curves to show the normal qPCR.

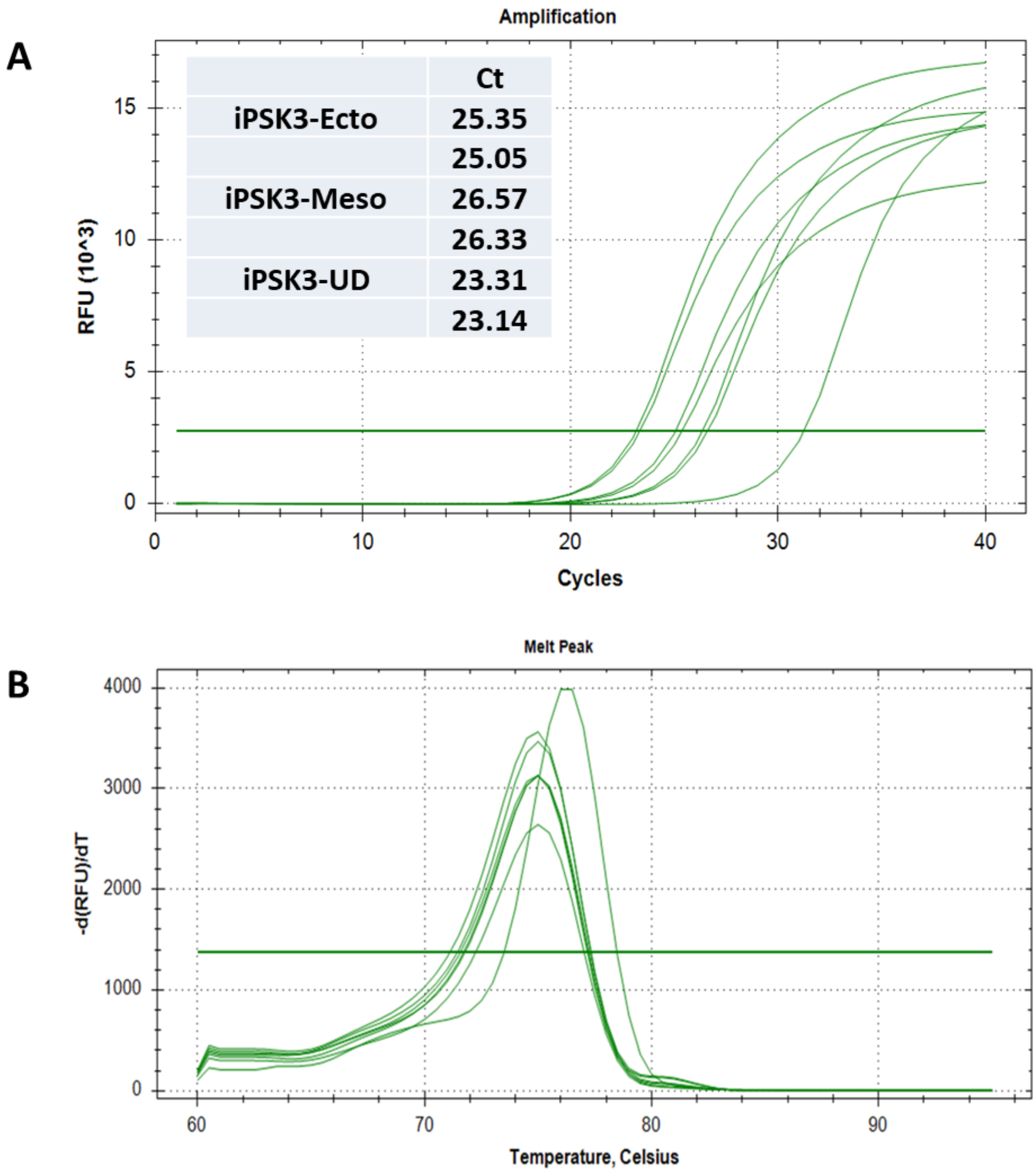

Supplementary Figure S8. Schematic illustration of crosstalk between miRNAs and canonical Wnt signaling. Adapted from Nie et al, 2018 (reference 86 in the main text).

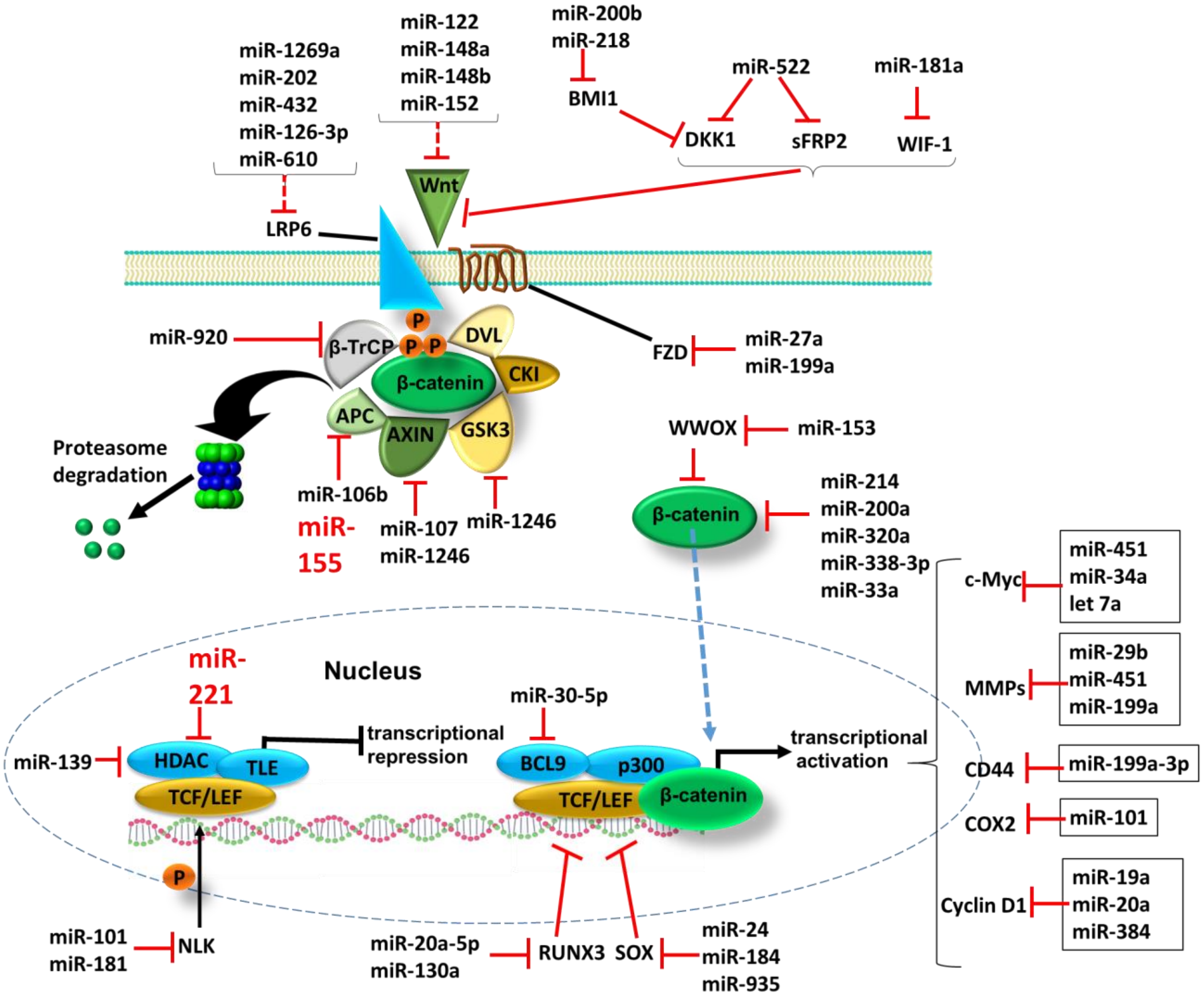

Supplementary Table S1. A list of antibodies.

Cells	Primary Antibody	Origin/ Isotype	Supplier/ Cat#	Dilution
Exosomes	CD63	Mouse IgG_1	Santa Cruz, SC-5275	1:100<br>1:1000 (WB)
	TSG101	Mouse IgG_1	Santa Cruz, SC-136111	1:100<br>1:1000 (WB)
	Alix	Mouse IgG_1	Santa Cruz, SC-49268	1:1000 WB
	Hsc70	Mouse IgG_{2a}	Santa Cruz, SC-7298	1:1000 WB
	CD81	Rabbit Polyclonal IgG	Santa Cruz, SC-9158	1:1000 WB
Negative exosome marker	Calnexin	Rabbit Polyclonal IgG	Santa Cruz, SC-11397	1:1000 WB
Proliferation	BrdU	Mouse IgG_1	Life Technologies, 03-3900	1:200
Mesoderm-cardiomyocytes	α-actinin	Mouse IgG_1	Sigma, A7811	1:800
	Nkx2.5	Rabbit Polyclonal IgG	Santa Cruz, sc-14033	1:400
Ectoderm-neural cells	Nestin	Rabbit IgG	Sigma, N5413	1:100
	Pax6	Mouse IgG_1	Santa Cruz, sc-81649	1:100
	β-tubulin III	Mouse IgG_1	Millipore, MAB1637	1:200
	Glutamate	Rabbit IgG	Sigma, G6642	1:1000
	GABA	Rabbit IgG	Sigma, A2052	1:1000
Secondary	Alexa 488, goat anti-mouse IgG_1	-	Life Technologies, A-21121	1:200
	Alexa 594, goat anti-rabbit IgG	-	Life Technologies, A-11012	1:400

Supplementary Table S2. NTA analysis for PEG isolation method.

Identifier	Mode particle size (nm)	Mean particle size (nm)	Standard deviation (nm)	Total particles (E8)/mL
Particle free PBS	240	188	8.6	0.12
iPSK3 undifferentiated EVs	134	209	8.9	26
iPSK3 cardiac mesoderm EVs	155	183	8.2	29
iPSK3 ectoderm EVs	148	187	8.5	35

Supplementary Table S3. Comparison of different house-keeping genes for miRNA RT-PCR.

Sample	House-keeping Gene	Ct value
Ecto EVs	univ-snord44	28.81
	univ-snord44	28.91
Meso EVs	univ-snord44	29.60
	univ-snord44	29.40
ipSK3 EVs	univ-snord44	27.56
	univ-snord44	27.56
Ecto EVs	univ-snord48*	26.15
	univ-snord48*	26.52
Meso EVs	univ-snord48	28.78
	univ-snord48	28.36
ipSK3 EVs	univ-snord48	27.03
	univ-snord48	26.81
Ecto EVs	U6 FR	19.39
	U6 FR	19.36
Meso EVs	U6 FR	20.50
	U6 FR	20.48
ipSK3 EVs	U6 FR	17.38
	U6 FR	17.47

* Bad melting curve.
